# Parameters and surrounding rock control of gob-side driving under double key stratum after roof cutting

**DOI:** 10.1038/s41598-024-55679-1

**Published:** 2024-03-01

**Authors:** Lei Xu, Yuzhao Ma, Davide Elmo, Shuxue Ding, Hengzhong Zhu, Honglin Liu, Wenfeng Li, Wen Chen

**Affiliations:** 1 School of Civil Engineering, Henan Polytechnic University, Jiaozuo, 454000 Henan China; 2 International Joint Research Laboratory of Henan Province for Underground Space Development and Disaster Prevention, Henan Polytechnic University, Jiaozuo, 454003 China; 3 The Norman B. Keevil Institute of Mining Engineering, University of British Columbia, Vancouver, V6T 1Z4 Canada; 4 College of Mining and Safety Engineering, Shandong University of Science and Technology, Qingdao, 266590 Shandong China; 5 School of Geology and Mining Engineering, Xinjiang University, Urumqi, 830046 Xinjiang China; 6 Earth and Environmental Sciences, Los Alamos National Laboratory, Los Alamos, NM 87545 USA; 7 LEM3 Laboratory, Université de Lorraine, 7 Rue Félix Savart, 57070 Metz, France

**Keywords:** Gob-side entry driving, Narrow coal pillar, Deviatoric stress, Roof cutting, Cantilever structure, Engineering, Civil engineering

## Abstract

Taking the return-airway 4204 with roof cutting in Longquan Coal Mine as the engineering background, roof structure, key parameters, and deviatoric stress evolution were studied. Conclusion: The Key Stratum within a 4–8 times mining height is considered as Near Key Stratum. Cutting the roof makes it possible to form a cantilever structure of the Key Stratum on the solid coal side, which is more conducive to the stability of gob-side roadway. During cutting angle of 90–55°, the deviatoric stress increases linearly, and the increase rate is coal pillar > solid coal > roof > floor. While cutting length from 0 to 35 m, the deviatoric stress decreases linearly, and the decreasing range: coal pillar > solid coal > roof > floor. When coal pillar width is from 30 to 4 m, the deviatoric stress of left side and floor presents a “single peak” distribution. The deviatoric stress of coal pillar changes from an asymmetric “double peak” to a bell-shaped distribution, and the deviatoric stress of roof changes from a “single peak” to an asymmetric “double peak” distribution. Under same coal pillar width, the deviatoric stress of left, coal pillar and roof after roof cutting decreases most obviously, followed by the floor. Finally, the coal pillar width is 8 m, the cutting angle is 75°, the cutting length is 20 m, and the hole spacing is 1.0 m. The support scheme is bolt + metal mesh + steel belt + anchor cable combined support. The stable period of roadway is about 10 days.

## Introduction

Gob-side roadway driving has been popularized in China for several decades. The traditional method is to leave 5–8 m narrow coal pillars to support the overlying strata and isolate the gob. However, this method is easy to cause severe roadway deformation characterized by strong floor heave and narrow coal pillar becoming inclined and moving inward^1–3^. As an essential work in coal mining, many scholars have carried out some research in the monitoring and evaluation system and achieved good results^4–8^. Reducing the width of the coal pillar and the deformation of the roadway along gob is the goal pursued by most scientific and technological personnel.

Regarding the surrounding rock structure, Wu et al.^9^ used FLAC3D software to simulate the layout of the cutting line of the gob-side entry retaining, and believed that the angle between the prefabricated roof cutting line and the vertical line of the roadway had little effect on the lateral displacement of the coal mining body in the gob-side entry retaining. When the roof cutting angle is 10°, the plastic range is small and the stability of surrounding rock structure is better. Sun et al.^10^ studied the influence of different roof cutting angles and roof cutting heights on the surrounding rock structure evolution of gob-side entry retaining. The results show that the supporting resistance required by the roof increases with the increase of roof cutting angle and height. Finally, they determined the roof cutting height is 8 m and the roof cutting angle is 15°. Guo et al.^11^ studied the fracture law of gob-side entry retaining roof through theoretical analysis and numerical simulation. They believed that the tensile strength of immediate roof and main roof is closely related to the fracture of gate roof, and the rotation and subsidence of main roof increases the deformation and failure of surrounding rock. The caving gravel cannot connect with the roof is the key to the instability of the surrounding rock structure. Khanal et al.^12^ employed site-specific geological and geotechnical information and longwall panel geometry to determine the appropriate chock capacity and believed that the massive roof strata had higher chock convergence than the bedded strata. Li et al.^13^ evaluated the Key Stratum’s fracture modes and the fractured block’s stability. They found that after the completion of the working face, the overlying rock will form a small supporting structure, a sizeable beam-like structure, and a macro compression arch structure from the bottom to the top. The small structure’s stability depended on a large structure's stability, which in turn depended on the stability of the macrostructure. Li et al.^14^ through field observation and numerical simulation, clearly showed that roadway failure was mainly due to main roof movement. Roof-cutting could enhance “macro-structure” formation and decrease the failure zone near the roadway. Roof-cutting could be used in production to reduce development time and increase productivity by blasting or hydraulic fracturing.

Regarding surrounding rock stress, He et al.^15^ established four FLAC^3D^ numerical models: large coal pillar, narrow coal pillar I, narrow coal pillar II, and no coal pillar. It is considered that the peak of lateral abutment pressure was the highest in large coal, followed by narrow coal pillars, and the lowest in non-coal pillar mining. Aiming at the problem of the premature collapse of rib or remnant pillars, Das et al.^16^ designed different underground structures and adequate support systems under complex geological conditions. The control measures were taken by setting up the optimal size of rib or remnant pillars in the goaf and installing good support systems in the working areas. Based on the stress distribution law study of narrow coal pillar, Yang et al.^17^ analyzed the stress and deformation law of surrounding rock under different coal pillar widths in the island working face of gob-side entry driving by numerical calculation, and determined that the reasonable width of coal pillar was 4 m. Qin et al.^18^ explored the mine pressure law of gob-side entry retaining in deep thin coal seam, and concluded that the stress field of working face is asymmetric. After roadway excavation, obvious stress redistribution characteristics are formed. The vertical and horizontal stress in the coal seam developed a U-shaped distribution. Wang et al.^19^ employed a geomechanical model to investigate the changes in abutment pressure at the solid coal and coal pillar sides during the working face's mining and roadway excavation stages. The result showed that coal entity peak stress with different coal pillar widths is more excellent than coal pillar. With the increase in pillar width, the coal entity is closer to the most prominent abutment pressure position, which is unsuitable for roadway support. Ptáček et al.^20^ investigated the magnitude of stress changes and principal axis direction changes. It is considered that the stress change is manifested as the change in stress magnitude and the rotation of the stress component axis. Rotations of the principal axes emerged as essential factors of the stress tensor changes and their influence on rock mass deformation and roadway stability. Xu et al.^21^ believed that due to the influence of strong mining, the deformation of gob-side roadway with fully mechanized caving mining was asymmetric. The uneven stress in the surrounding rock is the main reason for asymmetric deformation and failure.

Through the numerical model, Zhang et al.^22^ believed that the average stress in the coal pillar is more significant than in the panel rib, indicating that the coal pillar transmits more roof load. Roof pre-splitting treatment is good, but design parameters depend on geology and geotechnics. Zhang et al.^23^ showed that gob-side roadway control should consider the whole process of roadway support in the last sublevel. Han et al.^24^ used UDEC software to simulate and analyze the reasonable width of coal pillar in gob-side entry under multi-key stratum mining under high stress, and determined that 6 m width coal pillar is the inflection point of vertical stress, plastic zone and displacement distribution.

There is a tradition of protecting roadways with large coal pillars still prevalent in coal-producing areas in China like Shanxi, Shaanxi, and Inner Mongolia, which requires further research. With mine pressure understanding and roof-cutting technology, academic thought has changed from avoiding mine pressure to using it. Based on the return-airway 4204 (gob-side roadway) in Longquan Coal Mine, this paper discusses gob-side roadways’ cutting angle, cutting length, coal pillar width to deviatoric stress, and surrounding rock structure. It also does industrial tests.

## Project overview

### Mine location

The Longquan Coal Mine is situated in Loufan County, Shanxi Province, belonging to the southern area of the Ningwu coalfield. The geological condition of Longquan Coal Mine is simple, and the main coal-bearing strata are the Shanxi Formation of the Lower Permian and the Taiyuan Formation of the Upper Carboniferous. Currently.4# coal seam is mainly mined.

### Geological conditions and mining relationship

The 4# coal seam has a range of thickness from 6.2 to 7.4 m, and is usually 6.8 m thick (with a buried depth of around 455 m), so the entirety of the area is retrievable and secure. 4# coal seam is gas coal. The coal is black and glassy, with a brown streak, and a bright fissure.

**Figure 1:**
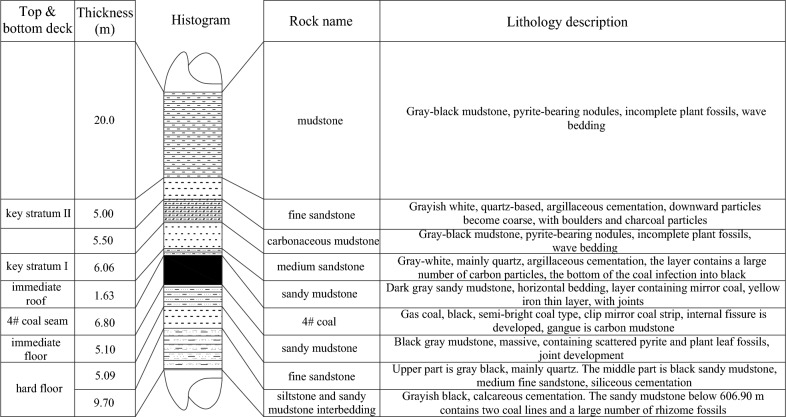
Stratum histogram.

The distribution of the rock stratum is shown in Fig. 1. The immediate roof is composed of sandy mudstone with a thickness of 0.5–2 m, with an average thickness of 1.63 m. It is deep gray with horizontal bedding and contains fossilized plant leaves. There are two Key Stratum from the bottom to the top. The medium sandstone (Key Stratum I) is 4.50–7.62 m thick, with an average thickness of 6.06 m, grey-white, and mainly quartz. Fine sandstone (Key Stratum II) is 4.5–9 m thick, with an average thickness of 5 m. 5.5 m-thick carbonaceous mudstone is between the two Key Stratum.

The immediate floor is sandy mudstone with a thickness of 3–15.1 m and an average thickness of 5.1 m, containing scattered pyrite and plant leaf fossils and joint development. The hard floor is 5.09 m fine sandstone; the thickness of siltstone and sandy mudstone interbedding is 7.40–12.00 m; the average thickness is 9.70 m, greyish black, calcareous cementation, hard.

**Figure 2:**
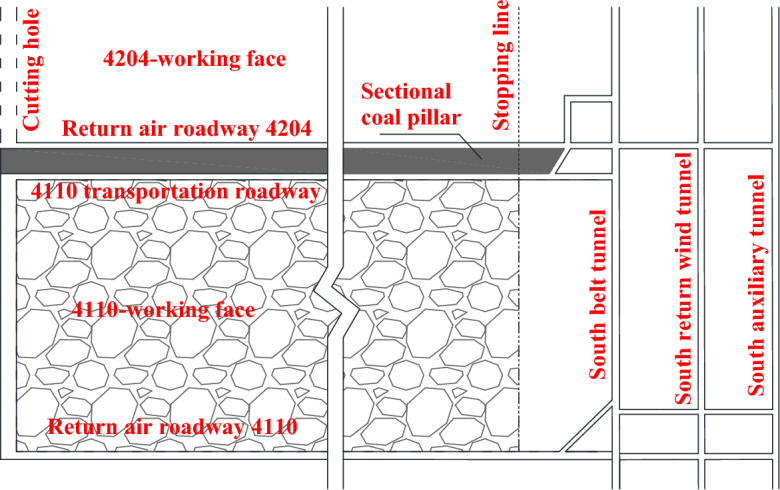
Mining relationship.

As shown in Fig. 2, after the 4110-working face (250 m wide) is mined out, it is planned to arrange the return airway of the 4204-working face (250 m wide) along the gob. As the top coal is broken, the return-airway 4204 is planned to be arranged along the roof. The mining method of the two working faces is top coal mining at all height at one time, where the coal cutting height is 3.5 m, and the coal caving height is 2.7–3.9 m.

## Design of surrounding rock structure of gob-side roadway by roof cutting

### Relationship between Key Stratum and caving zone height

According to Fig. 1, the medium sandstone (Key Stratum I) is located between 1.63 m and 7.69 m above the coal seam, and the fine sandstone (Key Stratum II) is located between 13.19 and 18.19 m above the coal seam.

1 $$ \begin{gathered} \subset \\ \Delta_{key} = 0\sim (4\sim 8)M \\ \supset \\ \end{gathered} $$

where: *M* is mining thickness, m; and Δ_key_ is the Key Stratum, m.

According to the classical mine pressure theory, the caving zone height on a longwall mining face is typically 4 to 8 times the height of the mining face. As shown in Eq. (1), when the Key Stratum is located within a range of 4 to 8 times the mining height, it is considered to be a “Near Key Stratum.” When the Key Stratum is located at the boundary of this range, it is considered to be a “critical Key Stratum.” When the Key Stratum is located outside of this range, it is considered to be a “remote Key Stratum”.

The total height of top coal mining at one time of 4110-working face is 6.80 m, and the recovery rate is calculated at 90%, which is only equivalent to the thickness of the mined coal body of 6.12 m. According to Eq. (1), the caving zone height of the 4110-working face is between 24.48–48.96 m. Even if the height of the caving zone is calculated according to the minimum mining height of four times, it can be judged that these two Key Stratums are in the caving zone, which will control the caving mode and pressure transmission law of the strata in the caving zone. The fracture mode and structure of these two Key Stratum will be the factors that influence the rock pressure of the gob-side roadway. Therefore, it is possible to modify the fracture mode of the Key Stratum by cutting the roof, thereby reducing the mine pressure in the gob-side roadway. There are several key parameters to consider, such as the position of the roof cutting line, the cutting angle, and the cutting length.

### Roof cutting position and cantilever structure

**Figure 3:**
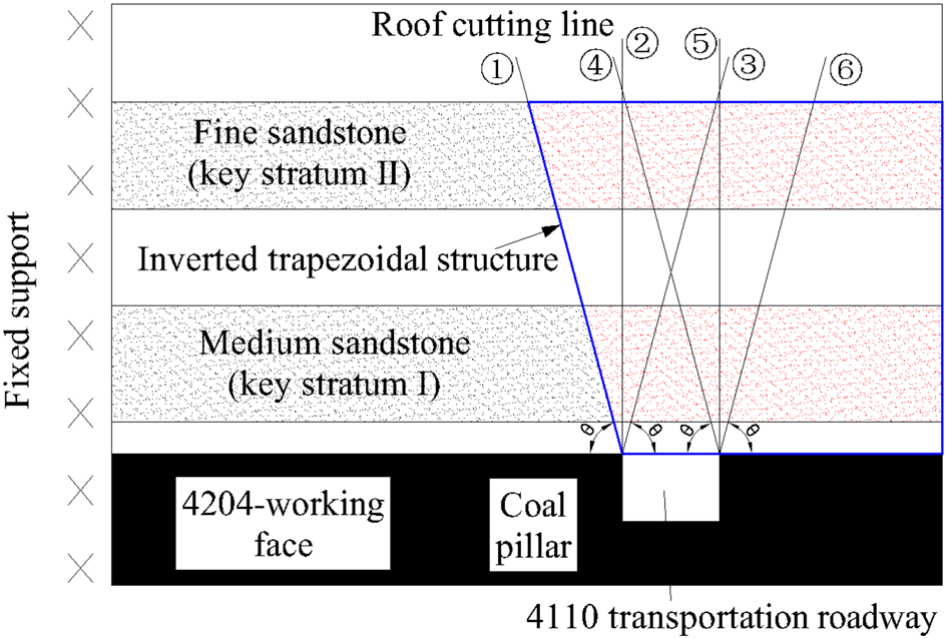
Different positions of roof cutting line.

As shown in Fig. 3, the different positions of the roof cutting line indicate the different fracture modes of the Key Stratum. Choosing roof-cutting lines ③ and ④ is not a good option as it cuts directly above the roadway and damages the stratum above it, violating the principle of protecting the integrity of surrounding rock in the roadway control concept of “NATM”. This can lead to the roof of 4110 transportation roadway falling prematurely. The roof cutting lines ② and ⑤ are located at the two shoulder angles of the 4110 transportation roadway, respectively, and are perpendicular to the roof. After the roof cutting, the integrity of the roadway roof is at risk as the roof is likely to slide along the cutting seam as a whole, which increases the risk of roadway maintenance. Therefore, this is not the best choice. The roof cutting line ① and ⑥ are both located in the stable area of the shoulder angle of the roadway, which helps in protecting the integrity of the roof above the roadway. After mining, the stratum will slide naturally along the roof-cutting line. However, the roof cutting line ⑥ will extend the cantilever structure of sandstone (Key Stratum I) and fine sandstone (Key Stratum II) in the roof, making the cantilever beam longer and increasing the weight it bears, resulting in stress concentration in the lower coal seam, which is not conducive to roadway stability. Consequently, line ① is the best choice, as it can not only shorten the extension length of the cantilever structure of the Key Stratum, but also ensure the integrity of the roof.

### Roof cutting length and double cantilever structure

**Figure 4:**
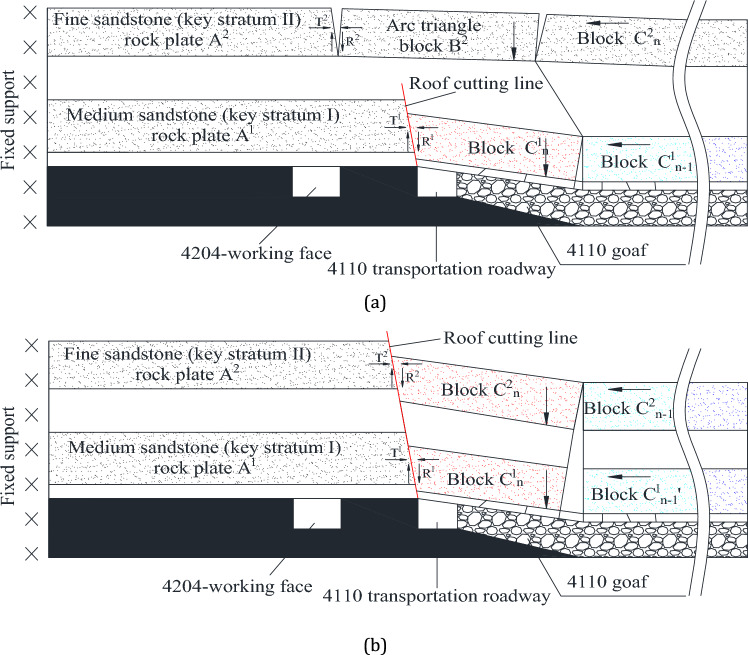
The rock structure of gob-side roadway with different cutting lengths: (**a**) Cutting off Key Stratum I; and (**b**) Cutting off Key Stratum I and II.

When the roof cutting line only cuts through the medium sandstone (Key Stratum I) as depicted in Fig. 4a, the medium sandstone (Key Stratum I) forms a cantilever structure rock plate A^1^. Under the influence of gravity, the Key Stratum I at the gob side is broken into multiple pieces. The rock block C^1^_n_ is superimposed on the rock plate A^1^. At this point, the fine sandstone (Key Stratum II) is not cut off and retains its traditional breaking pattern, resulting in the formation of A^2^, B^2^ (arc triangle block), and C^2^. The arc triangle block B^2^ forms a movable three-hinge arch structure with rock plate A^2^ and rock block C^2^. Then, the behaviour of the mine pressure in the gob-side roadway is regulated by the arc triangle block B^2^. It is clear that this structure will continue to have an impact on the lower coal seam, and it is not conducive to the stability of the gob-side roadway.

When the roof cutting line cuts through both the medium sandstone (Key Stratum I) and the fine sandstone (Key Stratum II), the breaking pattern of these two Key Stratums is completely altered, as seen in Fig. 4b. The fine sandstone (Key Stratum II) is transformed into a cantilever structural plate A^2^, and the rock block C^2^_n_ overlaps with the rock plate A^2^. Under the influence of gravity, it can smoothly slide towards the gob. The roof cutting altered the failure mechanism of the double Key Stratum, causing the absence of arc triangle blocks and resulting in a cantilever structure for the double Key Stratum. The rock slab that was cut at the gob side will slide down into the gob under the force of gravity. As a result, return-airway 4204 (gob-side roadway) is positioned beneath the double cantilever structure composed of Key Stratum I and II, which improves the stability of the gob-side roadway.

### Analysis of the cutting angle and S-R stability principle of composite rock block C_n_

The rock blocks C^1^_n_ and C^2^_n_ hold the weak middle layer (mudstone) together, forming the composite rock block C_n_. It forms a hinged relationship with the rock plates A^1^ and A^2^. To better utilize the mine pressure to make the composite rock block C_n_ slide and avoid the underlying strata bearing more rock weight, it is necessary to design the cutting angle reasonably.

**Figure 5:**
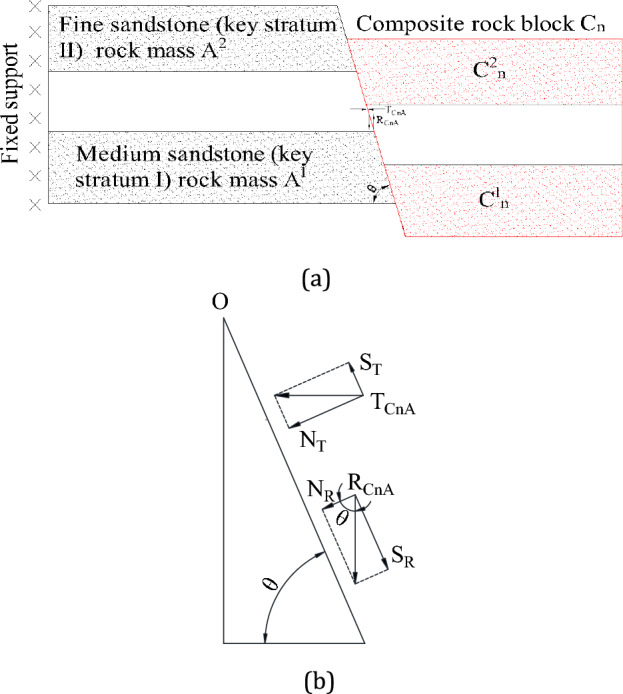
Cutting surface stability model of S-R theory: (**a**) cutting surface force diagram; and (**b**) mechanical equilibrium decomposition diagram of cutting surface.

Without considering the failure of the roof-cutting surface caused by a slip, it is considered that the internal friction angle of the roof-cutting surface is a constant value. According to Mohr–Coulomb theory and the S-R stability principle. The stable relationship between composite rock block C_n_ and rock slabs A^1^ and A^2^ is shown in Fig. 5. The friction force on a roof-cutting surface is the product of the normal stress on that surface and the coefficient of friction (the tangent value of cohesion), which helps prevent the block from sliding. It acts as a counterforce with the shear stress on the roof cutting surface, as shown in Eq. (2).

2 $$ \begin{gathered} \left( {N_{R} + N_{T} } \right)\tan \varphi \begin{array}{*{20}c} < \\ = \\ > \\ \end{array} S_{R} - S_{T} \hfill \\ \begin{array}{*{20}c} {} & {} & \Downarrow \\ \end{array} \hfill \\ \left( {R_{{C_{n} A}} \cos \theta + T_{{C_{n} A}} \sin \theta } \right)\tan \varphi \begin{array}{*{20}c} < \\ = \\ > \\ \end{array} R_{{C_{n} A}} \sin \theta - T_{{C_{n} A}} \cos \theta \hfill \\ \end{gathered} $$

where *T*_CnA_ is the horizontal force of the composite rock block C_n_ acting on the cutting surface; *R*_CnA_ is the vertical force of the composite rock block C_n_ acting on the cutting surface; *θ* is the angle between the cutting surface and the vertical direction (55–90°); and *φ* is internal friction angle (38–45°).

Transform Eq. (2) to derive Eq. (3).

3 $$ {\raise0.7ex\hbox{${R_{{C_{n} A}} }$} \!\mathord{\left/ {\vphantom {{R_{{C_{n} A}} } {T_{{C_{n} A}} }}}\right.\kern-0pt} \!\lower0.7ex\hbox{${T_{{C_{n} A}} }$}}\begin{array}{*{20}c} > \\ = \\ < \\ \end{array} \cot \left( {\theta { - }\varphi } \right) $$

When the left side of Eq. (3) is larger than the right side, the composite block C_n_ slides; when the left side is equal to the right side, the composite block C_n_ is in a critical state; when the left side is smaller than the right side, the composite block C_n_ is stable. When the roof cutting angle is closer to 90° (the roof cutting line is perpendicular to the roof), the smaller the value of the right side of Eq. (3), the smaller the vertical load force *R*_CnA_ of the composite block on the cutting surface, the smaller the normal stress component *R*_CnA_cosθ + *T*_CnA_sinθ on the roof cutting surface, and the smaller the generated friction force. At this point, it is most beneficial for the composite block C_n_ to slide down.

In combination with the analysis in Sect. "Relationship between Key Stratum and caving zone height", it can be observed that the roof-cutting line has a slight inclination towards the solid coal side, which favours the formation of an “inverted trapezoidal” roof-cutting structure for the roadway roof. Considering the scheme in which the roof cutting line slightly inclines to the solid coal side, it can not only promote the sliding of the composite block C_n_ after mining, but also ensure the stability of the roadway before mining.

## Numerical model of gob-side roadway with roof cutting

### Model parameters

The numerical model is established with 3DEC 5.2. The dimensions of the model are: 100 m (length) × 5 m (width) × 64.88 m (height). The bottom, front, back, left, and right boundaries of the model are displacement boundaries, and the top is a stress boundary (with an equal weight of rock). The Mohr–Coulomb model was adopted for the rock block, and the Coulomb slip model was adopted for the joint. Based on the test results of the basic mechanical properties of the surrounding rock, the parameters of each stratum and contact surface are determined, as shown in Table 1 and Table 2.

**Table 1:** Mechanics parameters of each stratum.

Rock stratum	Thickness/m	Bulk/GPa	Shear/GPa	Density /kg·m^−3^	Cohesion/MPa	Tensile/MPa	Friction/(°)	Dilation/(°)
Mudstone	27.2	3.68	2.10	2360	2.10	1.50	25	1
Fine sandstone	5.09	9.91	4.92	2750	3.26	2.19	30	3
Carbonaceous mudstone	3.63	3.68	2.10	2360	2.10	1.50	25	1
Medium sandstone	6	7.87	3.38	2700	3.26	2.19	28	3
Sandy mudstone	5.1	3.68	2.10	2360	2.10	1.50	25	1
4# coal seam	7.78	2.35	1.47	1350	1.50	1.10	20	1
Sandy mudstone	5.1	3.68	2.10	2360	2.10	1.50	25	1
Fine sandstone	10.5	8.82	4.84	2750	3.30	2.47	30	3
Aluminiferous mudstone	5.7	3.68	2.10	2360	2.10	1.50	25	1
Siltstone and sandy mudstone interbedding	2	7.52	3.15	2720	3.20	2.30	29	3

**Table 2:** Mechanics parameters of the joint surface.

Rock stratum joint	Normal stiffness/GPa	Shear stiffness/GPa	Cohesion/MPa	Friction/(°)	Tensile/MPa	Dilation/(°)
Mudstone	3.60	2.70	1.20	15	0.50	0
Fine sandstone	7.10	3.40	1.30	20	0.47	1
Carbonaceous mudstone	3.60	2.70	1.20	15	0.50	0
Medium sandstone	7.40	3.50	1.76	20	0.50	1
Sandy mudstone	3.60	2.70	1.20	15	0.50	0
4# coal seam	2.00	1.80	1.10	10	0.10	0
Sandy mudstone	3.60	2.70	1.20	15	0.50	0
Fine sandstone	7.20	3.38	1.26	18	0.39	1
Siltstone and sandy mudstone interbedding	6.80	3.30	1.36	18	0.39	1

### Simulation scheme

Rectangular section of the return-airway 4204: width × height: 5.5 m × 3.8 m. The parameters of the anchor bolt and anchor cable are presented in Table 3.

**Table 3:** Parameters of the anchor bolt and anchor cable in 3DEC.

Name	Roof spacing /mm	Spacing between sides /mm	Length /mm	Diameter /mm	Density /kg· m^−3^	Elastic modulus /GPa	Tensile strength /kN	Bonding stiffness /N·m^-2^	Bonding strength /N·m^−1^	Prestress /kN
Bolt	850	850	2400	22	7500	200	120	2e6	4e5	80
Anchor cable	1100		8300	22	7800	300	300	2e6	4e5	100

**Figure 6:**
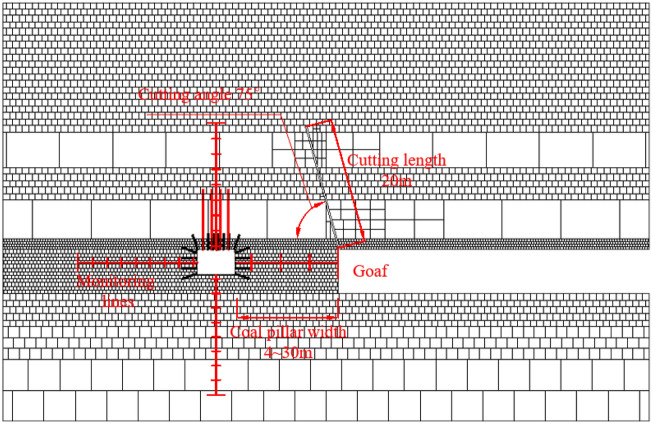
Numerical model of a gob-side roadway with cutting angle 75°, cutting length 20 m, coal pillar width 8 m.

The key parameters of roof cutting include roof cutting angle, cutting length, and different coal pillar widths (roof cutting or no cutting). Other simulation schemes are outlined in Table 4, Table 5, and Table 6. There are a total of 38 models. This section only lists this one numerical model, as shown in Fig. 6.

**Table 4:** Simulation scheme of different cutting angles.

Coal pillar width 8 m and cutting length 20 m								
Cutting angle/°	90	85	80	75	70	65	60	55

**Table 5:** Simulation scheme of different cutting lengths.

Coal pillar width 8 m and cutting angle 75°								
Cutting length/m	0	5	10	15	20	25	30	35

**Table 6:** Simulation scheme of different coal pillar widths.

Cutting length 20 m, cutting angle 75° and different coal pillar widths/m											
No roof cutting	30	25	20	18	16	14	12	10	8	6	4
Roof cutting	30	25	20	18	16	14	12	10	8	6	4

### Physical meaning of deviatoric stress

In China, abutment pressure is commonly used as an indicator of mine pressure, however, it does not take into account the relationship among various stresses. It is the alteration of the relationship among stresses that leads to the failure of rocks. Is there a physical quantity that can reflect both the stress state of the rock and the relationship between various forces? Deviatoric stress can effectively reconcile the above two factors.

**Figure 7:**
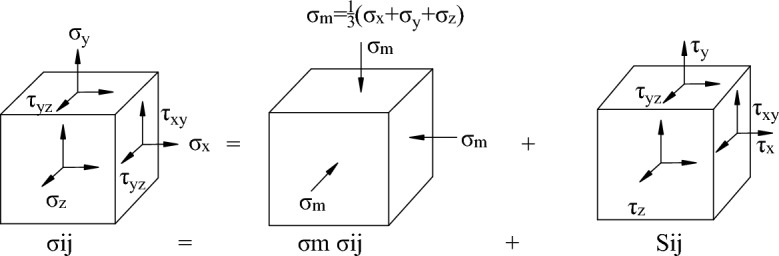
Stress state decomposition at any point.

The stress state at any point in a rock medium is illustrated in Fig. 7. It can be decomposed into two parts: the spherical stress tensor and the deviatoric stress tensor. The mathematical expressions for these two tensors are given.

4 $$ \sigma_{i} { = }\left[ {\begin{array}{*{20}c} {\sigma_{1} } & 0 & 0 \\ {} & {\sigma_{2} } & 0 \\ {SYM} & {} & {\sigma_{3} } \\ \end{array} } \right] = \left[ {\begin{array}{*{20}c} {\sigma_{m} } & 0 & 0 \\ {} & {\sigma_{m} } & 0 \\ {SYM} & {} & {\sigma_{m} } \\ \end{array} } \right] + \left[ {\begin{array}{*{20}c} {S_{1} } & 0 & 0 \\ {} & {S_{2} } & 0 \\ {SYM} & {} & {S_{3} } \\ \end{array} } \right]\; $$

**Figure 8:**
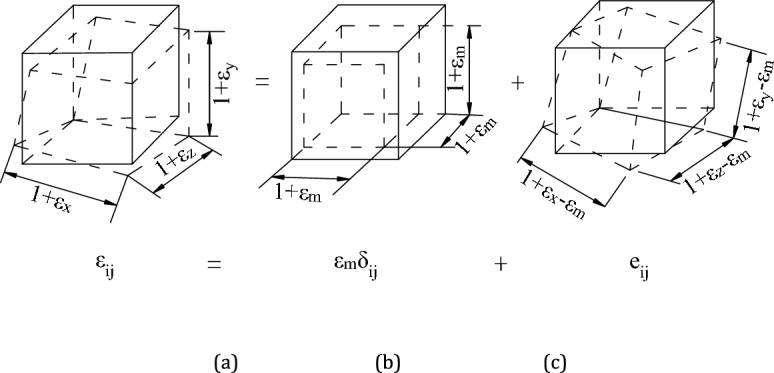
Decomposition of deformation. (**a**) changes in volume and shape; (**b**) changes in volume and (**c**) changes in shape.

Deformation in a rock medium is classified into two types: volume change (as shown in Fig. 8b) and shape change (as shown in Fig. 8c). The spherical stress tensor is responsible for changes in volume, while the deviatoric stress tensor causes changes in the material's shape. These shape changes ultimately led to the deformation and failure of the rock (as shown in Fig. 8 a).

In Eq. (4), *σ*_m_ is the spherical stress, calculated as *σ*_m_ = (*σ*_1_ + *σ*_2_ + *σ*_3_)/3 in MPa; *S*_i_ represents the main deviatoric stress, calculated as *S*_i_ = *σ*_i_-*σ*_m_ in MPa. Among them, the first principal deviatoric stress *S*_1_ plays a significant role in the distortion and fracture of rock, and is often referred to as deviatoric stress. It is the chosen measurement index in this study.

## Deviatoric stress of the gob-side roadway affected by cutting angle

According to the simulation scheme in Table 4, the fixed coal pillar width is 8 m, and the roof cutting length is 20 m. The influence of different cutting angles (90°– 55°) on the deviatoric stress of the gob-side roadway is discussed.

### Deviatoric stress distribution influenced by cutting angle

**Figure 9:**
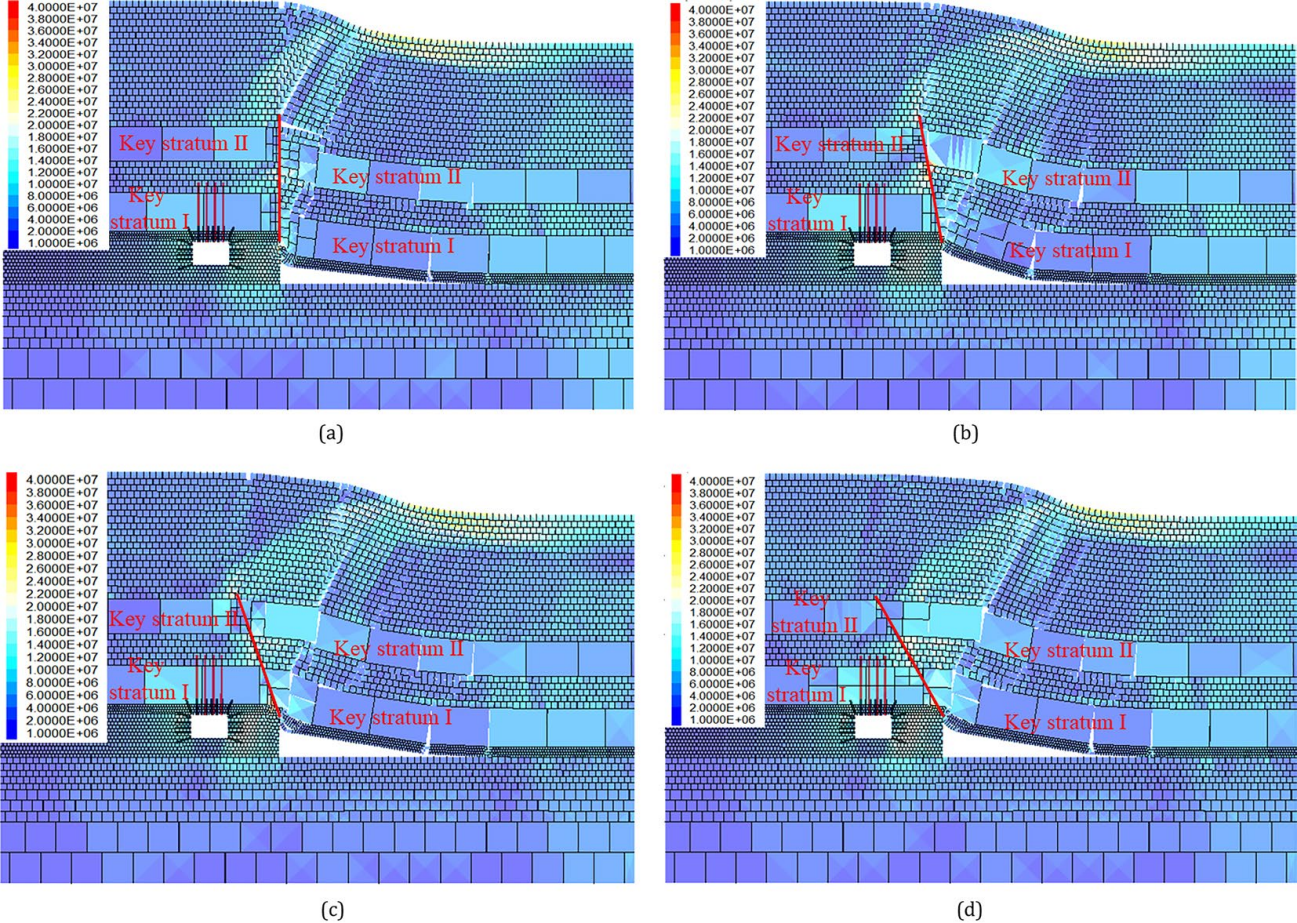
Deviatoric stress cloud map of gob-side roadway with various cutting angles: (**a**) cutting angle 90°; (**b**) cutting angle 80°; (**c**) cutting angle 70° and (**d**) cutting angle 60°.

As illustrated in Fig. 9, the deviatoric stress is observed to be concentrated primarily at the head of the roof-cutting line and subsequently transferred to deeper regions within the rock stratum. The closer the roof-cutting line is to the side of the roadway (return-airway 4204), the more pronounced the stress concentration in the surrounding rock becomes. From the perspective of the surrounding rock structure, the smaller the cutting angle, the less sufficient the collapse of the key rock stratums in the mined area, resulting in an increase in the weight of the overlying rock layers transmitted to the underlying rock layers, and subsequently leading to an increase in the degree of deviatoric stress concentration in the underlying coal layers.

**Figure 10:**
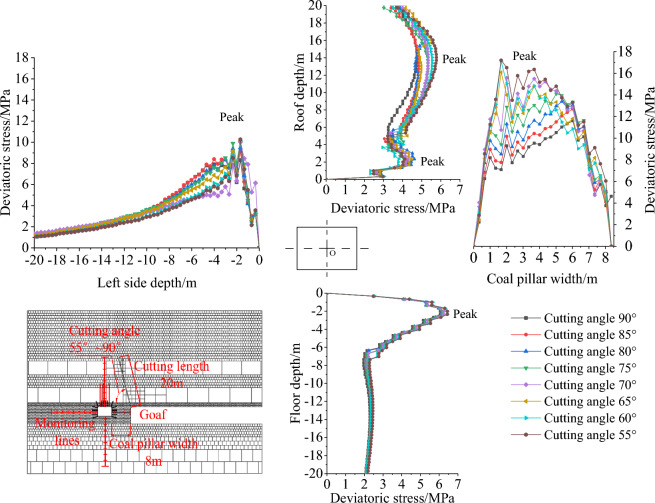
Deviatoric stress distribution of gob-side roadway with different cutting angles.

Extract the deviatoric stress values on the roof, floor, and sides of the survey line, as represented in Fig. 10. As the cutting angle is decreased from 90 to 55°, the deviatoric stress on the left side of the roadway (solid coal) and the floor shows a single peak distribution, with the peak located at approximately 2 m. The deviatoric stress on the right side of the roadway (coal pillar) shows a bell-shaped distribution, with the peak value located approximately in the middle of the coal pillar. The deviatoric stress on the roof shows an asymmetric double-peak distribution, with the shallow peak located at approximately 2 m depth and the deeper peak located at approximately 14 m depth. The deeper peak is greater than the shallow peak. As the cutting angle decreases, the peak values of deviatoric stress gradually increase, but there is no obvious change in the peak position.

### Vertical comparison of cutting angle effect on the deviatoric stress peak value

To determine the effect of the cutting angle on the maximum deviatoric stress, the maximum values of the deviatoric stress should be compared with that of a cutting angle of 90° and calculated using the Eq. (5).

5 $$ Increases_{\alpha } = \frac{{Deviatoric\,stress_{{Cutting\,angle_{x} }} - Deviatoric\,stress_{{Cutting\,angle_{{90^{^\circ } }} }} }}{{Deviatoric\,stress_{{Cutting\,angle_{{90^{^\circ } }} }} }} $$

**Figure 11:**
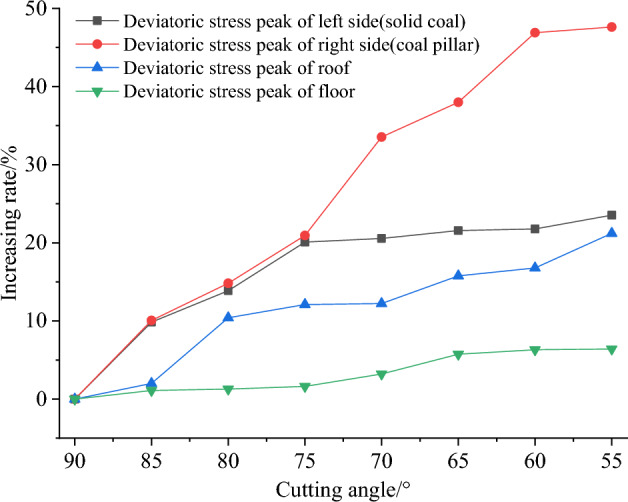
The increase of deviatoric stress peak value affected by cutting angle.

The results can be found in Table 7 and Fig. 11. When the cutting angle is 90°, the peak values of deviatoric stress on the left side, right side (coal pillar), roof, and floor of the roadway are 8.31 MPa, 11.61 MPa, 4.76 MPa, and 6.06 MPa, respectively. When the cutting angle is 75°, the peak values of deviatoric stress on the left side, right side (coal pillar), roof, and floor of the roadway are 9.98 MPa, 14.10 MPa, 5.33 MPa, and 6.16 MPa, respectively, which are an increase of 20.09%, 21.44%, 12.10%, and 1.62% respectively compared to the case when the cutting angle is 90 degrees. When the cutting angle is 55°, the peak deviatoric stress of the left side, the right side (coal pillar), the roof, and the floor are 10.27 MPa, 17.21 MPa, 5.77 MPa, and 6.44 MPa respectively. The peak deviatoric stress of the left side, the right side (coal pillar), the roof and the floor are increased by 23.54%, 48.22%, 21.22%, and 6.40% respectively, as shown in Fig. 11. The growth is roughly linear.

**Table 7:** Comparison of the deviatoric stress peak values of gob-side roadway with different cutting angles.

Cutting angle/°	Deviatoric stress of left side (solid coal)		Deviatoric stress of right side (coal pillar)		Deviatoric stress of roof		Deviatoric stress of floor	
	Peak value/MPa	Amplification/%	Peak value/MPa	Amplification/%	Peak value/MPa	Amplification/%	Peak value/MPa	Amplification/%
90	8.31	0.00	11.61	0.00	4.76	0.00	6.06	0.00
85	9.13	9.85	12.83	10.51	4.85	2.01	6.12	1.10
80	9.46	13.87	13.39	15.30	5.25	10.40	6.13	1.29
75	9.98	20.09	14.10	21.44	5.33	12.10	6.16	1.62
70	10.02	20.55	15.57	34.09	5.34	12.22	6.25	3.21
65	10.10	21.57	16.09	38.56	5.51	15.77	6.40	5.73
60	10.12	21.78	17.13	47.49	5.56	16.78	6.44	6.32
55	10.27	23.54	17.21	48.22	5.77	21.22	6.44	6.40

As analyzed above, it can be inferred that the best unloading effect is achieved when the cutting angle is 90° (perpendicular to the roof). During the cutting angle range of 90– 55°, the peak value consistently exhibits the characteristic of the right side (coal pillar) > left side (solid coal) > floor > roof. As a result of decreasing the cutting angle, the transfer of weight from the rock layer downwards is intensified, leading to a concomitant elevation in the magnitude of stress concentration and peak values of the underlying rock stratum. From the perspective of amplification, the characteristics are right side (coal pillar) > left side (solid coal) > roof > floor. This implies that increasing the cutting angle can effectively improve the stress environment of the lower gob-side roadway, with the most significant improvement observed on the right side (coal pillar) and left side (solid coal) side.

## Deviatoric stress of the gob-side roadway affected by cutting length

### Deviatoric stress distribution influenced by cutting length

Based on the simulation scenario presented in Table 5, with a fixed cutting angle of 75° and coal pillar width of 8 m, the evolution of the deviatoric stress in the surrounding rock of the gob-side roadway (return-airway 4204) is studied when the cutting length varies from 0 to 35 m.

**Figure 12:**
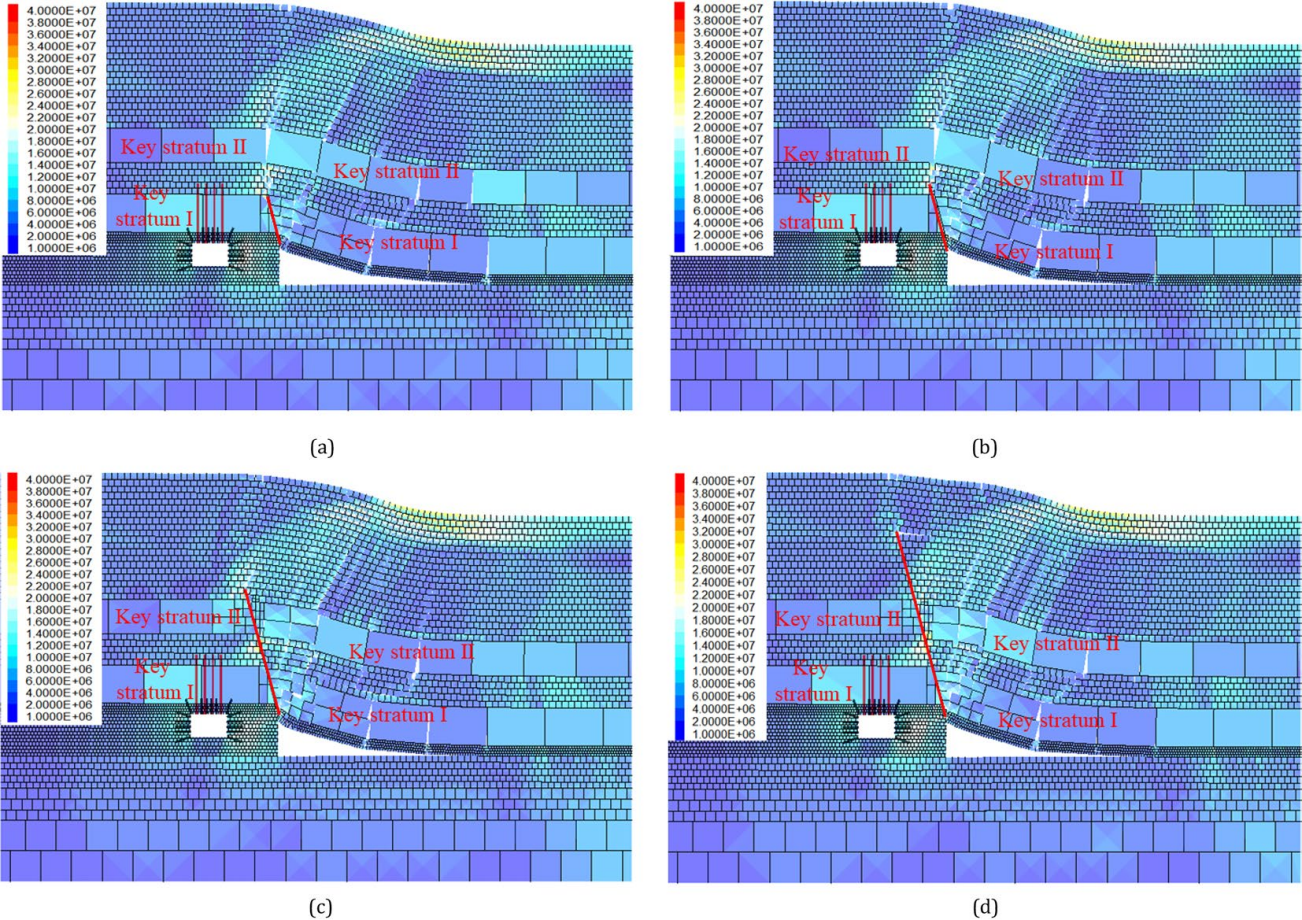
Deviatoric stress cloud map of gob-side roadway with various cutting lengths: (**a**) cutting length of 5 m; (**b**) cutting length of 10 m; (**c**) cutting length of 20 m and (**d**) cutting length of 30 m.

When the cutting length is 5–10 m, only the immediate roof and the medium sandstone (Key Stratum I) are cut off. Fine sandstone (Key Stratum II) can still form a hinged structure at the edge of the gob, which can transfer the weight of the overlying strata to the underlying coal seam. When the cutting length is greater than 20 m, the fine sandstone (Key Stratum II) is also cut off. At this point, the double Key Stratum on the solid coal side becomes a double cantilever structure. The increase in cutting length leads to a more complete collapse of the rock layers on the gob side, as illustrated in Fig. 12, which manifests itself as a reduction in the degree of stress concentration of the deviatoric stress. The deviatoric stress is concentrated at the head of the cutting line. With the increase of the cutting length, the deviatoric stress is gradually transferred to the deeper rock layers.

**Figure 13:**
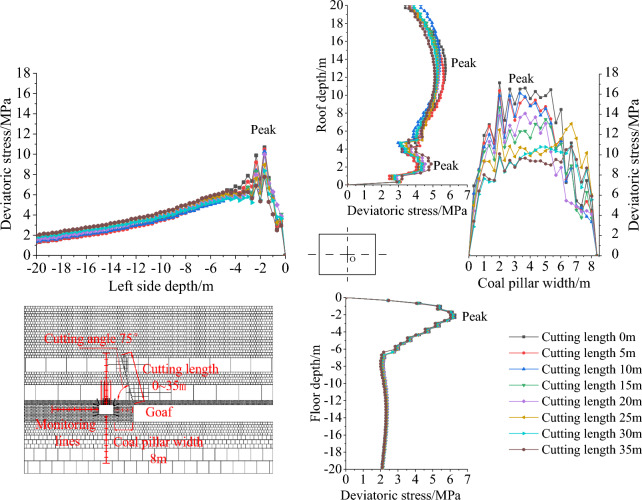
Deviatoric stress distribution of gob-side roadway with different cutting lengths.

Extract the deviatoric stress on the roof, floor, and both sides of the survey line, as shown in Fig. 13, and its distribution is similar to those of the cutting angle in the previous section. The details are as follows: In the process of cutting the roof from 0 to 35 m, the deviatoric stress on the left side (solid coal) and the floor of the roadway both show a “single peak” distribution, and the peak position is around 2 m deep. The deviatoric stress on the right side of the roadway (coal pillar) is bell-shaped, and the peak value is approximately located in the middle of the coal pillar. The peak value of deviatoric stress on the roof shows an asymmetric “double-peak” distribution, with the shallow peak located around 2 m deep in the roof and the deep peak located around 14 m deep in the roof, with the deep peak being larger than the shallow peak. As the length of the roof cutting increases, the peak values of the deviatoric stress gradually decrease, but the position of the peak values does not change significantly.

### Vertical comparison of cutting length effect on the deviatoric stress peak value

To quantify the effect of the cutting length on the peak value of deviatoric stress, the peak values are compared against the deviatoric stress of the cutting length of 0 m, respectively, and calculated using the Eq. (6).

6 $$ Reduction_{L} = \frac{{Deviatoric\,stress_{{Cutting\,length_{x} }} - Deviatoric\,stress_{{Cutting\,length_{0m} }} }}{{Deviatoric\,stress_{{Cutting\,length_{0m} }} }} $$

**Figure 14:**
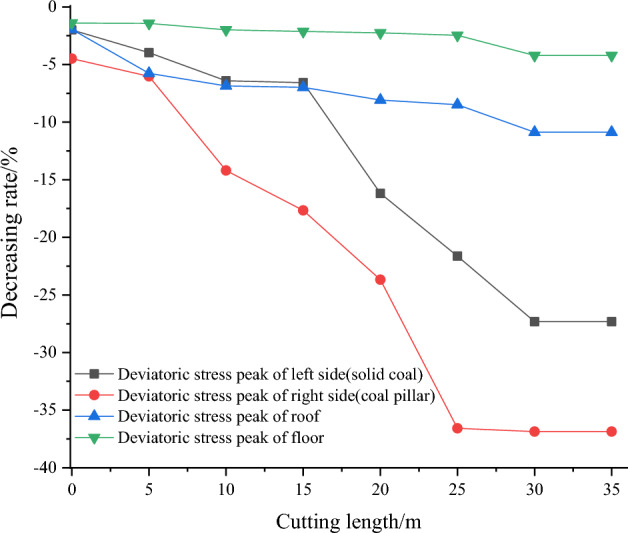
The reduction of deviatoric stress peak value affected by cutting length.

The results are shown in Table 8 and Fig. 14. When the roof cutting length is 0 m, the deviatoric stress peaks on the left side, right side (coal pillar), roof, and floor of the roadway are 10.68 MPa, 17.13 MPa, 5.73 MPa, and 6.29 MPa, respectively. When the cutting length is 20 m (the cutting line passes through the Key Stratum II), the peak values of deviatoric stress on the left side, right side (coal pillar), roof, and floor of the roadway are 9.98 MPa, 14.10 MPa, 5.33 MPa, and 6.16 MPa respectively. These values represent reductions of 6.58%, 17.66%, 6.98%, and 2.12%, respectively, compared to the deviatoric stress peaks at a cutting length of 0 m. When the cutting length is 35 m, the peak values of deviatoric stress on the left, right side (coal pillar), roof, and floor are 7.77 MPa, 10.81 MPa, 5.11 MPa, and 6.02 MPa respectively. Compared to the deviatoric stress peaks at a cutting length of 0 m, these values represent reductions of 27.32%, 36.86%, 10.87%, and 4.21% respectively, as shown in Fig. 14. The deviatoric stress peak values decrease in a roughly linear manner.

**Table 8:** Comparison of the deviatoric stress peak values of gob-side roadway with different cutting lengths.

Cutting length/m	Deviatoric stress of left side (solid coal)		Deviatoric stress of right side (coal pillar)		Deviatoric stress of roof		Deviatoric stress of floor	
	Peak value /MPa	Amplification/%	Peak value/MPa	Amplification/%	Peak value /MPa	Amplification/%	Peak value/MPa	Amplification/%
0	10.68	0.00	17.13	0.00	5.73	0.00	6.29	0.00
5	10.47	− 2.00	16.36	− 4.49	5.62	− 1.94	6.20	− 1.40
10	10.26	− 3.97	16.09	− 6.03	5.40	− 5.77	6.20	− 1.43
15	10.00	− 6.41	14.69	− 14.20	5.34	− 6.86	6.16	− 1.99
20	9.98	− 6.58	14.10	− 17.66	5.33	− 6.98	6.16	− 2.12
25	8.95	− 16.19	13.07	− 23.68	5.27	− 8.08	6.15	− 2.25
30	8.37	− 21.63	10.86	− 36.58	5.25	− 8.49	6.13	− 2.46
35	7.77	− 27.32	10.81	− 36.86	5.11	− 10.87	6.02	− 4.21

From the analysis above, it can be seen that the longer the cutting length is, the better the pressure relief effect is. When the cutting line is less than 15 m (before crossing the Key Stratum II), the peak value of deviatoric stress decreases gradually. When the cutting length is greater than 15 m, the rate of decrease in the peak value of deviatoric stress increased significantly. As the cutting length reaches 25 m, the rate of decrease in the peak deviatoric stress slows down. In the process of cutting length of 0–35 m, from the peak value, the peak values consistently display the characteristics of the right side (coal pillar) > left side (solid coal) > floor > roof. The increase of the cutting length cuts off the hanging plates of the two Key Stratum at the gob side, and the degree of deviatoric stress concentration of the lower strata was reduced. From the perspective of amplitude reduction, it is characterized by the right side (coal pillar) > left side (solid coal) > roof > floor. This shows that reasonable cutting length can effectively improve the stress environment of the lower gob-side roadway, and the most obvious improvement effect is on the right side (coal pillar) and left side (solid coal).

## Deviatoric stress of the gob-side roadway affected by pillar width

### Deviatoric stress distribution influenced by pillar width

Table 6 simulation scheme is adopted to study the evolution of deviatoric stress of gob-side roadway with different pillar widths under the conditions of no roof cutting and roof cutting (length 20 m, angle 75°).

**Figure 15:**
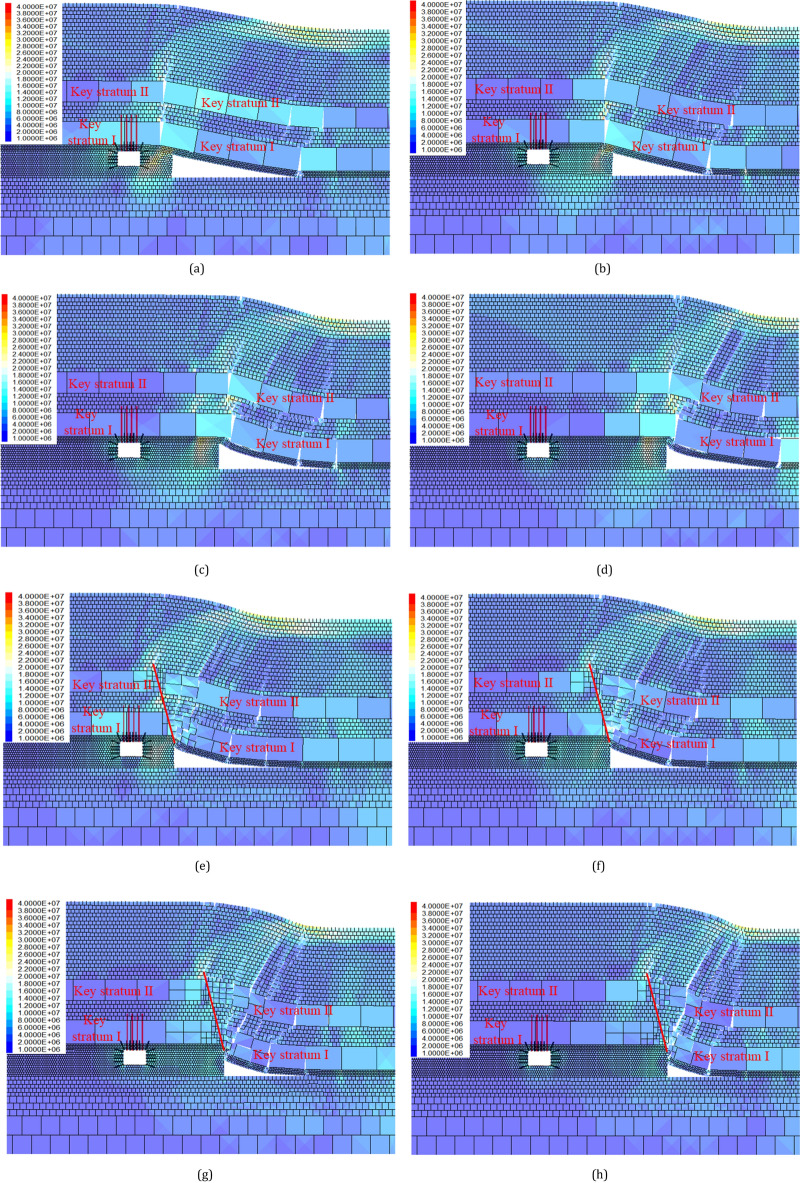
Cloud images of deviatoric stress of gob-side roadway with different pillar widths under no cutting or cutting (20 m, 75): (**a**) pillar width 8 m (no cutting); (**b**) pillar width 16 m (no cutting); (**c**) pillar width 20 m (no cutting); (**d**) pillar width 30 m (no cutting); (**e**) pillar width 8 m (cutting); (**f**) pillar width 16 m (cutting); (**g**) pillar width 20 m (cutting); and (**h**) pillar width 30 m (cutting).

As depicted in Fig. 15a–d, without roof cutting, when the previous working face (4110) is mined out, the overlying medium sandstone (Key Stratum I) and fine sandstone (Key Stratum II) form a hinge structure at the edge of the gob, supporting the overlying rock stratum while transferring stress downward. The wider the coal pillar, the further the gob is from the gob-side roadway (return-airway 4204), and the lower the deviatoric stress of the surrounding rock of the gob-side roadway (return-airway 4204). However, this improvement in stress conditions along the gob-side roadway (return-airway 4204) comes at the cost of increasing the width of the coal pillar.

When the previous working face (4110) is mined and the roof is cut, the caving mode of the two Key Stratum is changed, and leading to a concentration of stress at the head of the cutting line which is subsequently transferred to the deeper zone of the stratum. As illustrated in Fig. 15e–h, the collapse of the strata on the gob side is more pronounced, thus reducing the proportion of the weight of the overlying strata transmitted downwards through the articulated structure. Through vertical comparison, it can be observed that when the coal pillar width is the same, the concentration degree of deviatoric stress under roof-cutting conditions is significantly lower than that under non-roof-cutting conditions. For example, when the width of the coal pillar is 16 m, the concentration degree of the deviatoric stress in the surrounding rock of the roadway under the condition of roof cutting (Fig. 15b) is significantly lower than that under the condition of no roof cutting (Fig. 15f).

**Figure 16:**
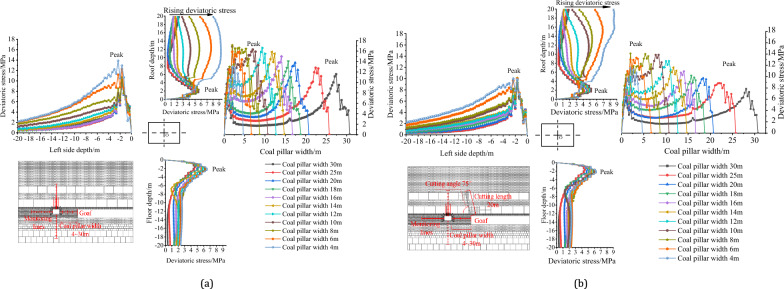
Deviatoric stress distribution of gob-side roadway with different pillar widths under no cutting or cutting: (**a**) non-roof cutting and; (**b**) roof cutting.

The deviatoric stress of the survey line, as shown in Fig. 16a,b, is similar in distribution patterns when the roof is not cut and when it is cut.

The distribution of left side (solid coal) deviatoric stress is “single peak” shaped, and as the width of the coal pillar decreases from 30 to 4 m, the position of the peak deviatoric stress on the left side does not change significantly, remaining around 2.0 m in depth, but the peak value gradually increases.

The deviatoric stress distribution of coal pillar: when the width of the coal pillar is greater than 10 m, the deviatoric stress is in the shape of an “asymmetric saddle”, which shows that the deviatoric stress on the gob side is higher than that on roadway side. When the width of the coal pillar is 10–4 m, the deviatoric stress of the coal pillar is bell-shaped, and the peak value is roughly located in the middle of the coal pillar. As the width of the coal pillar decreases, the peak value of deviatoric stress first increases and then decreases.

The distribution of roof deviatoric stress: When the coal pillar width is greater than 10 m, the deviatoric stress is approximately in the shape of a “single peak,” with the peak of deviatoric stress located around 2 m deep in the roof. As the width of the coal pillar decreases from 10 to 4 m, the deviatoric stress in the deep part of the roof sharply increases and gradually transits to an asymmetric “double peak” shape.

The distribution of floor deviatoric stress: the deviatoric stress is roughly in the shape of a “single peak,” with the peak of deviatoric stress around 2 m deep in the floor. As the width of the coal pillar decreases from 35 to 4 m, the distribution of shear stress does not change significantly. The position of the peak remains relatively stable.

### Vertical comparison of pillar width effect on the deviatoric stress peak value

With the decrease in coal pillar width, it is impossible to share more deviatoric stress caused by strata movement, and the gob-side roadway (return-airway 4204) is closer to the gob. To quantify the influence of coal pillar width on the peak value of deviatoric stress, the peak values of deviatoric stress are compared with that of coal pillar width of 30 m, respectively, and calculated with the Eq. (7).

7 $$ Vertical\,comparison_{pillar} = \frac{{Deviatoric\,stress_{{Coalpillar\,width_{x} }} - Deviatoric\,stress_{{Coalpillar\,width_{30m} }} }}{{Deviatoric\,stress_{{Coalpillar\,width_{30m} }} }} $$

**Figure 17:**
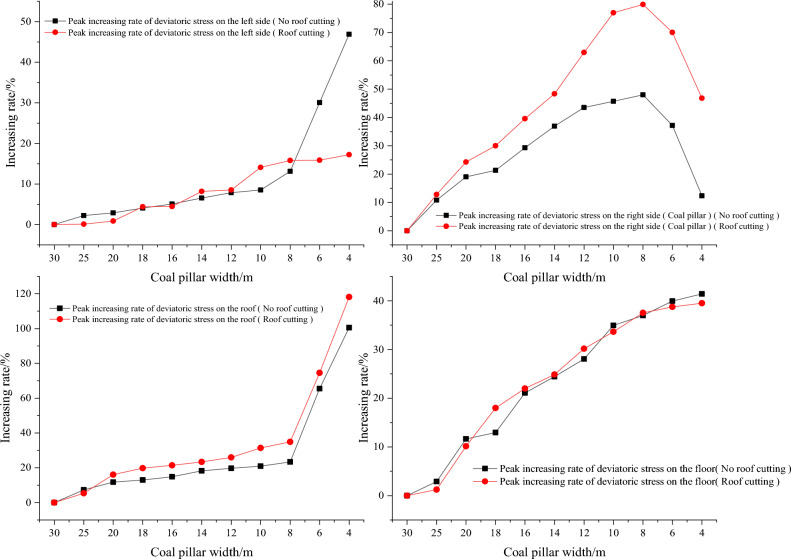
Variation of deviatoric stress peak value of gob-side roadway with different pillar widths.

The results are shown in Table 9, Table 10 and Fig. 17. When the width of the coal pillar is from 30 to 4 m, the deviatoric stress of the surrounding rock increases. From the perspective of peak development, the left side and floor increase in a linear way; the right side (coal pillar) increases first and then decreases, and the roof increases in a jumping way. In terms of growth rate, when the roof is not cut off, the peak value of deviatoric stress of the left side increases by 46.91%, that of the right side (coal pillar) by 48.00%, that of the roof by 100.50%, and that of the floor by 41.40%. After roof cutting, the peak value of the deviatoric stress of the left side increased by 0–17.22%, that of the right side (coal pillar) increased by 0–79.91%, that of the roof increased by 0–118.15%, and that of the floor increased by 0–39.49%. Regardless of whether the roof is cut off or not, the reduction of the coal pillar width will seriously affect the deviatoric stress in the surrounding rock of the gob-side roadway, especially the roof and the right side (coal pillar).

**Table 9:** Comparison of the deviatoric stress peak values of gob-side roadway with different pillar widths without cutting.

Coal pillar width/m	Deviatoric stress of left side (solid coal)		Deviatoric stress of right side (coal pillar)		Deviatoric stress of roof		Deviatoric stress of floor	
	Peak value/MPa	Amplification/%	Peak value/MPa	Amplification/%	Peak value/MPa	Amplification/%	Peak value/MPa	Amplification/%
30	9.45	0.00	11.57	0.00	4.65	0.00	4.59	0.00
25	9.66	2.22	12.82	10.79	4.98	7.26	4.72	2.89
20	9.72	2.90	13.77	19.03	5.19	11.73	5.13	11.64
18	9.83	4.09	14.04	21.33	5.25	13.00	5.19	12.95
16	9.93	5.09	14.96	29.31	5.34	14.85	5.56	21.09
14	10.06	6.54	15.84	36.91	5.50	18.28	5.71	24.41
12	10.19	7.87	16.60	43.50	5.56	19.73	5.88	28.05
10	10.25	8.54	16.86	45.69	5.62	20.92	6.20	34.94
8	10.68	13.11	17.13	48.00	5.73	23.36	6.29	36.96
6	12.29	30.07	15.87	37.16	7.69	65.45	6.42	39.92
4	13.88	46.91	13.00	12.35	9.32	100.50	6.49	41.40

**Table 10:** Comparison of the deviatoric stress peak values with different pillar widths after roof cutting.

Coal pillar width/m	Deviatoric stress of left side (solid coal)		Deviatoric stress of right side (coal pillar)		Deviatoric stress of roof		Deviatoric stress of floor	
	Peak value/MPa	Amplification/%	Peak value/MPa	Amplification/%	Peak value/MPa	Amplification/%	Peak value/MPa	Amplification/%
30	8.62	0.00	7.84	0.00	3.95	0.00	4.48	0.00
25	8.63	0.12	8.84	12.78	4.16	5.30	4.53	1.25
20	8.69	0.87	9.74	24.27	4.59	16.08	4.93	10.13
18	9.00	4.39	10.19	29.98	4.74	19.76	5.28	17.98
16	9.00	4.46	10.94	39.57	4.80	21.40	5.46	21.98
14	9.32	8.17	11.63	48.34	4.88	23.36	5.59	24.85
12	9.35	8.51	12.77	62.94	4.98	25.94	5.83	30.16
10	9.83	14.08	13.87	76.94	5.20	31.37	5.98	33.64
8	9.98	15.80	14.10	79.91	5.33	34.85	6.16	37.51
6	9.99	15.86	13.33	70.05	6.90	74.55	6.21	38.74
4	10.10	17.22	11.51	46.82	8.63	118.15	6.24	39.49

### Horizontal comparison of deviatoric stress peak value with no roof cutting and roof cutting

After cutting, the deviatoric stress of the surrounding rock is reduced. To quantitatively evaluate the reduction under the same coal pillar width, Eq. (8) is used for calculation.

8 $$ Horizontal\,comparison_{Pillar} = \frac{{Deviatoric\,stress(Roof\,cutting)_{{Coalpillar\,width_{x} }} - Deviatoric\,stress(No\,roof\,cutting)_{{Coalpillar\,width_{x} }} }}{{Deviatoric\,stress(No\,roof\,cutting)_{{Coal\,pillar\,width_{x} }} }} $$

The results are presented in Table 11. From the range of reduction, the peak value of deviatoric stress on the left side is reduced by 4.09–27.19%, that on the right side (coal pillar) is reduced by 11.48–32.26%, that on the roof is reduced by 6.98– 16.46%, and that on the floor is reduced by 0.11–4.07%. It can be observed that under the same width of a coal pillar, the deviatoric stress peak values of the left side (solid coal), right side (coal pillar), and roof are reduced significantly after roof cutting, while the deviatoric stress peak value of the floor only reduces by a few percent. Therefore, it can be deduced that roof cutting can significantly reduce the peak values of deviatoric stress in the gob-side roadway (return-airway 4204), particularly for the left side (solid coal), right side (coal pillar), and roof.

**Table 11:** Horizontal comparison of the deviatoric stress peak value of gob-side roadway with different coal pillar widths without roof cutting or roof cutting.

Coal pillar width/m	Peak deviatoric stress of left side/MPa			Peak deviatoric stress of right side (coal pillar)/MPa			Peak deviatoric stress of roof			Peak deviatoric stress of floor		
	No roof cutting	Roof cutting	Horizontal reduction/%	No roof cutting	Roof cutting	Horizontal reduction/%	No roof cutting	Roof cutting	Horizontal reduction /%	No roof cutting	Roof cutting	Horizontal reduction/%
30	9.45	8.62	− 8.74	11.57	7.84	− 32.26	4.65	3.95	− 14.90	4.59	4.48	− 2.52
25	9.66	8.62	− 10.71	12.82	8.84	− 31.04	4.98	4.16	− 16.46	4.72	4.53	− 4.07
20	9.72	8.69	− 10.55	13.77	9.74	− 29.28	5.19	4.59	− 11.59	5.13	4.93	− 3.83
18	9.83	9.00	− 8.47	14.04	10.19	− 27.44	5.25	4.74	− 9.81	5.29	5.28	− 0.11
16	9.93	9.00	− 9.29	14.96	10.94	− 26.89	5.34	4.80	− 10.05	5.56	5.46	− 1.80
14	10.06	9.32	− 7.35	15.84	11.63	− 26.61	5.50	4.88	− 11.24	5.71	5.59	− 2.17
12	10.19	9.35	− 8.20	16.60	12.77	− 23.09	5.56	4.98	− 10.48	5.88	5.83	− 0.91
10	10.25	9.83	− 4.09	16.86	13.87	− 17.73	5.62	5.20	− 7.55	6.20	5.98	− 3.45
8	10.68	9.98	− 6.58	17.13	14.10	− 17.66	5.73	5.33	− 6.98	6.29	6.16	− 2.12
6	12.29	9.99	− 18.71	15.87	13.33	− 16.02	7.69	6.90	− 10.22	6.42	6.21	− 3.33
4	13.88	10.10	− 27.19	13.00	11.51	− 11.48	9.32	8.63	− 7.41	6.49	6.24	− 3.83

## Control principle and key parameters of the gob-side roadway by roof cutting and surrounding rock

### Roof cutting control principle of the gob-side roadway

According to the traditional theory of gob-side entry driving in China, after the previous working face (4110) is mined, the main roof collapses and forms three blocks A, B (arc triangle block), and C. The gob-side entry driving is carried out beneath the arc triangle block B, and the blocks B, A, and C form a three-hinged arch structure. The stability of the arc triangle block B plays a crucial role in determining the stability of the lower gob-side roadway.

**Figure 18:**
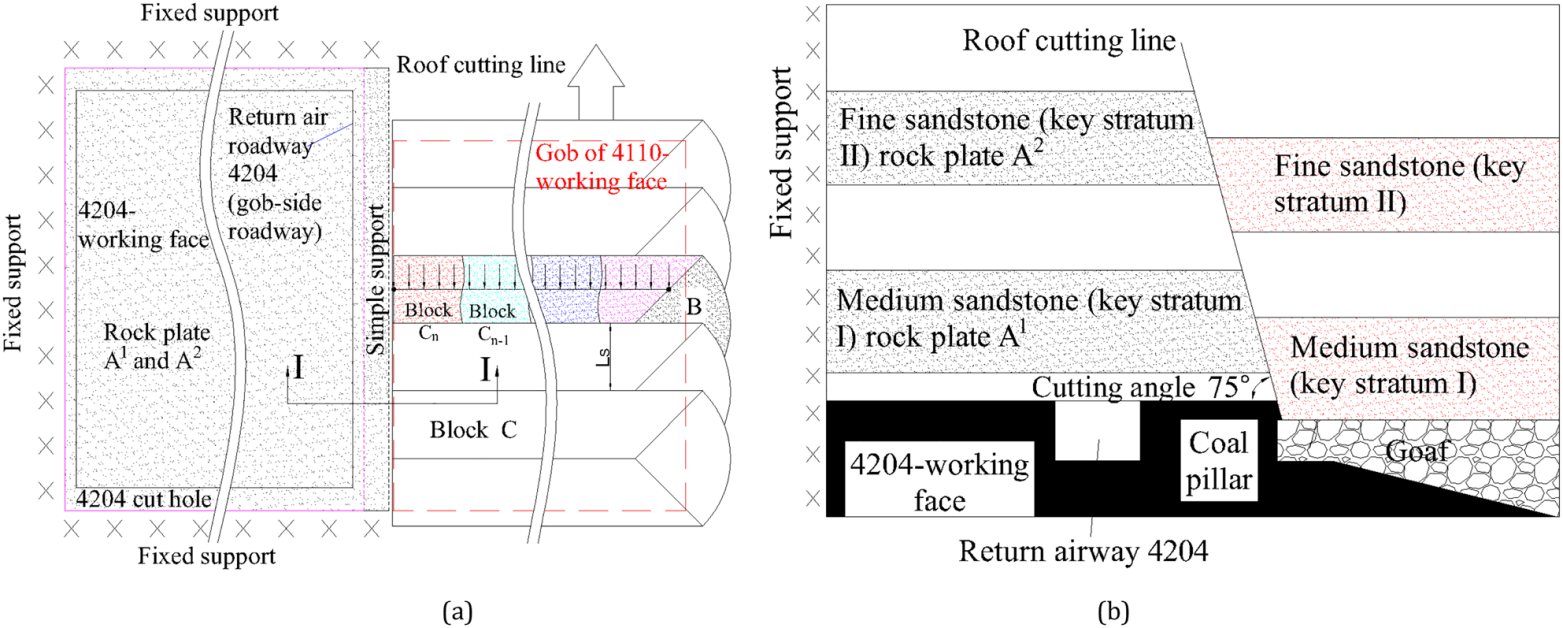
Surrounding rock control of the gob-side roadway by roof cutting: (**a**) planar graph; and (**b**) I-I section.

Roof-cutting technology can alter the fracture pattern of the Key Stratum. As shown in Fig. 18a, the Key Stratum above the 4204 working face is relatively intact after the roof cutting (at the roof cutting line position) of the 4110transportation roadway. At this time, the medium sandstone (Key Stratum I) and fine sandstone (Key Stratum II) above the 4204-working face are in a condition of three-sided fixed support and one-side simple support. As seen in section I-I (Fig. 18b), the return-airway 4204 (gob-side roadway) is under the protection of the cantilever beam composed of the complete rock slabs A^1^ and A^2^. With the advance of the previous working face (4110), the medium sandstone (Key Stratum I) and fine sandstone (Key Stratum II) are cut into composite rock slabs C, with a strike of Ls (periodic pressure step), a dip of Lc, and a thickness of h. The composite rock slab C can be regarded as a rock beam that is simply supported at both ends. Under the influence of overlying rock layers, it is likely to break in its middle first, resulting in rock slabs C_L_ and C_R_. Under the effect of gravity, these slabs will continue to be pulled apart, forming shorter rock blocks as shown in Fig. 18b. These rock blocks interlock with each other and sink together. The gob-side roadway (return-airway 4204) will be excavated under the structure of a double cantilever beam rock slab.

### Key parameters for roof cutting and surrounding rock control

As can be seen from the above analysis, the width of the coal pillar has the greatest impact on the deviatoric stress of the gob-side roadway, followed by the length and angle. As a result, the gob-side roadway with roof cutting should be designed in this order.

Coal pillar width: When the coal pillar width is less than 8 m, the deviatoric stress on the coal pillar is exactly “bell-shaped”, the deviatoric stress peak of the right side (coal pillar) continues to increase, while the deviatoric stress peak of the roof, floor and left side also continues to increase. When the width of the coal pillar increases to 10 m, the deviatoric stress on the coal pillar becomes a “saddle” shape, indicating that an elastic core has been formed in the middle part. 4# coal is 6.8 m thick, which is a thick coal seam. From the analysis of the cross-sectional shape of the coal pillar, when the width of the coal pillar is less than 8 m, the cross-sectional shape of the coal pillar gradually changes from rectangle to square to vertical rectangle, making it more difficult for the central elastic core to form. Therefore, the width of the coal pillar is designed to be 8 m.

Roof cutting length: When the cutting length passes through medium sandstone (Key Stratum I) and fine sandstone (Key Stratum II), the deviatoric stress in the surrounding rock can be greatly reduced. The reduction of deviatoric stress would not be significant by increasing the cutting length, and it would also increase the cost, so the cutting length is set at 20 m.

Roof cutting angle: While cutting at 90° produces the most effective relief effect, this contradicts NATM's idea of protecting the surrounding rock. Additionally, the roof cutting position is located in the advanced part of the working face, where dynamic pressure is prone to causing the premature collapse of the roof. When cutting at 75°, the down-propagated deviatoric stress is slightly increased by the overlying rock stratum, but the increase rate it is not significant. Moreover, the 75° slit makes the roof strata in an “inverted trapezoidal”, which can be temporarily installed on the roadway side. It will fall after the cut rock stratum enters the mined-out area. This is more conducive to maintaining the advanced roadway. As shown in Fig. 18b, the roof cutting angle of 75° is selected.

### Scheme of accumulative blasting

**Figure 19:**
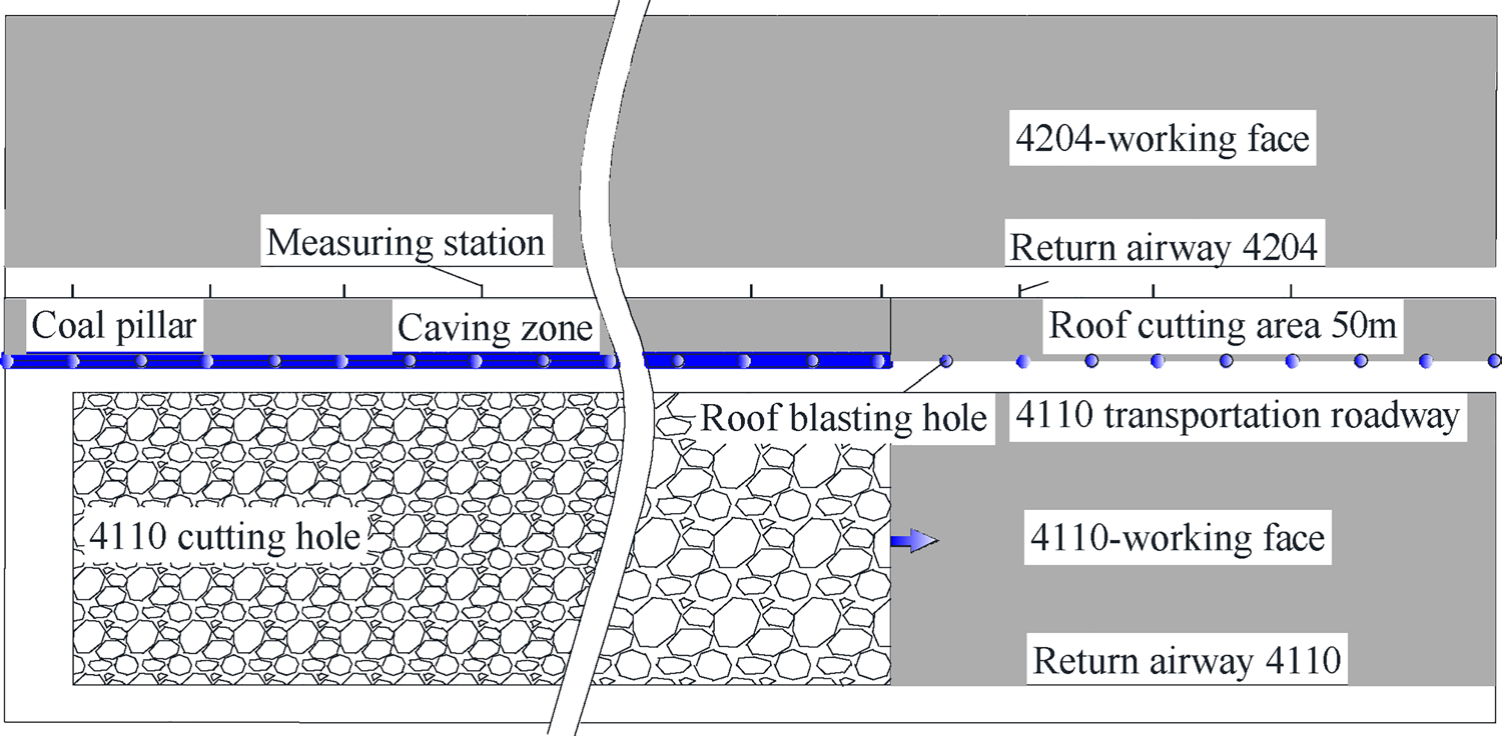
Layout of roof cutting.

Roof cutting location: Completed before the advance support of the working face, about 40– 50 m ahead of the working face, as shown in Fig. 19.

Borehole diameter: 48 mm.

The hole spacing is determined based on the blasting crack damage criterion, and the formula^25,26^ is as follows:

9 $$ d \le 2r_{b} \left[ {\frac{{\lambda p_{b} }}{{\left( {1 - D_{0} } \right)\sigma_{t} + p}}} \right]\frac{1}{\delta } $$

where: *d* is center distance of the pre-split hole, m; *r*_b_ is radius of pre-splitting hole, m; *λ* is lateral pressure coefficient, *λ* = *μ*/(1-*μ*); *σ*_t_ is dynamic tensile strength of rock mass, MPa; *p*_b_ is peak pressure of blast wave on the borehole side, MPa; *p* is horizontal in-situ stress, MPa; *δ* is attenuation coefficient of blasting stress wave, *δ* = 2*μ*/(1-*μ*); *μ* is Poisson's ratio; and *D*_0_ is initial rock damage parameter.

**Figure 20:**
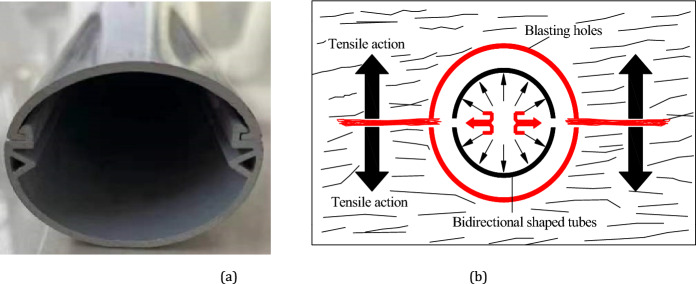
Accumulative blasting: (**a**) tube of accumulative blasting; and (**b**) diagram of accumulative blasting action.

The accumulative blasting is adopted, as shown in Fig. 20. Compared to fine sandstone (Key Stratum II), the medium sandstone (Key Stratum I) has a higher strength. Therefore, based on the fine sandstone, the dynamic tensile strength *σ*_t_ = 50 MPa. The vertical stress is calculated as 9.4 MPa based on a depth of 455 m and an average rock density of 2650 kg/m3. Considering the lateral pressure coefficient of 1.1, the *p* is calculated as 10.35 MPa. Rock Poisson’s ratio* μ* = 0.25. The rock damage parameter value *D*_0_ = 0.5, according to the distribution of cracks in the medium sandstone (Key Stratum I). According to the experimental results of accumulative blasting, the maximum blasting pressure at the pre-cutting slit is taken as *p*_b_ = 1500 MPa, the borehole radius is *r*_b_ = 24 mm, and it can be calculated that *d*≈1.01 m. Meanwhile, according to field test results, the final spacing of the cut-off holes is determined as 1 m. This can be adjusted according to the changes in the roof properties and field test.

Other parameters: The diameter of the cut-off hole is 48 mm. The distance between the hole to the side is 200 mm. A continuous coupled forward double-stage loading structure with a radial uncoupling coefficient of 1.3 and a sealing length of 1/3 was chosen.

## Analysis of support scheme and implementation effect of gob-side roadway

### Support scheme of gob-side roadway

**Figure 21:**
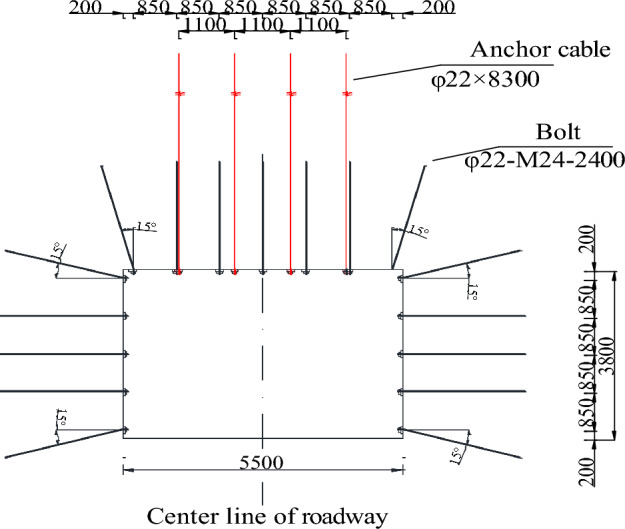
Support parameters.

Combined support scheme: bolt + metal mesh + steel belt + anchor cable. Details are shown in Fig. 21.

- (1) Roof bolt and layout.
- φ22-M24-2400 high-strength left-handed spiral steel bolt without longitudinal reinforcement,7 in each row. The inter-row spacing is 850 mm × 900 mm. The bolts at the two shoulder angles are inclined outward by 15°. Each bolt is equipped with one K2335 and one Z2360 anchoring agent. The high-strength arch-shaped tray has dimensions of 150 mm x 150 mm x 10 mm, and the arch height is no less than 36 mm. It is equipped with a spherical pad and a damping washer ring. The pre-tightening torque shall not be less than 300N·m.
- Roof steel strip: BHW4.5–280-8–5300;
- Mesh specification:8# wire double-layer diamond-shaped metal mesh, mesh hole 50 mm × 50 mm, mesh sheet size 5500 mm × 1100 mm.
- The overlap width shall not be less than 100 mm, double-strand 16# wire connection, double wire double buckle, ten buckles per meter, and the number of twists shall not be less than 3 rounds.
- (2) Roof anchor cable and layout.
- φ22 × 8300 mm (19 cores) high-extensibility, inter-row spacing of 1100 mm × 1800 mm (4–0-4 interval layout), vertical to the roof. Each anchoring cable is equipped with one K2335 and two Z2360 anchoring agents. The tray is a 300 mm × 300 mm × 16 mm high-strength adjustable center support plate with an arch height not less than 60 mm. The pre-tension force is 200 kN.
- (3) Two-side bolts and layout.
- φ22-M24-2400 high-strength left-handed spiral steel bolt without longitudinal reinforcement, 5 in each row. The inter-row spacing is 850 mm × 900 mm. The bolts at the shoulder angle and bottom angle are inclined outward by 15°. Each bolt is equipped with one K2335 and one Z2360 anchoring agent. The high-strength arch-shaped tray has dimensions of 150 mm x 150 mm x 10 mm, and the arch height is no less than 36 mm. It is equipped with a spherical pad and a damping washer ring. The pre-tightening torque shall not be less than 300N·m.
- Two sides steel strip: BHW4.5–280-8–5300;
- Mesh specification:8# wire double-layer diamond-shaped metal mesh, mesh hole 50 mm × 50 mm, mesh sheet size 5500 mm × 1100 mm. The overlap width shall not be less than 100 mm, double-strand 16# wire connection, double wire double buckle, ten buckles per meter, and the number of twists shall not be less than 3 rounds.

### Analysis of implementation effect

**Figure 22:**
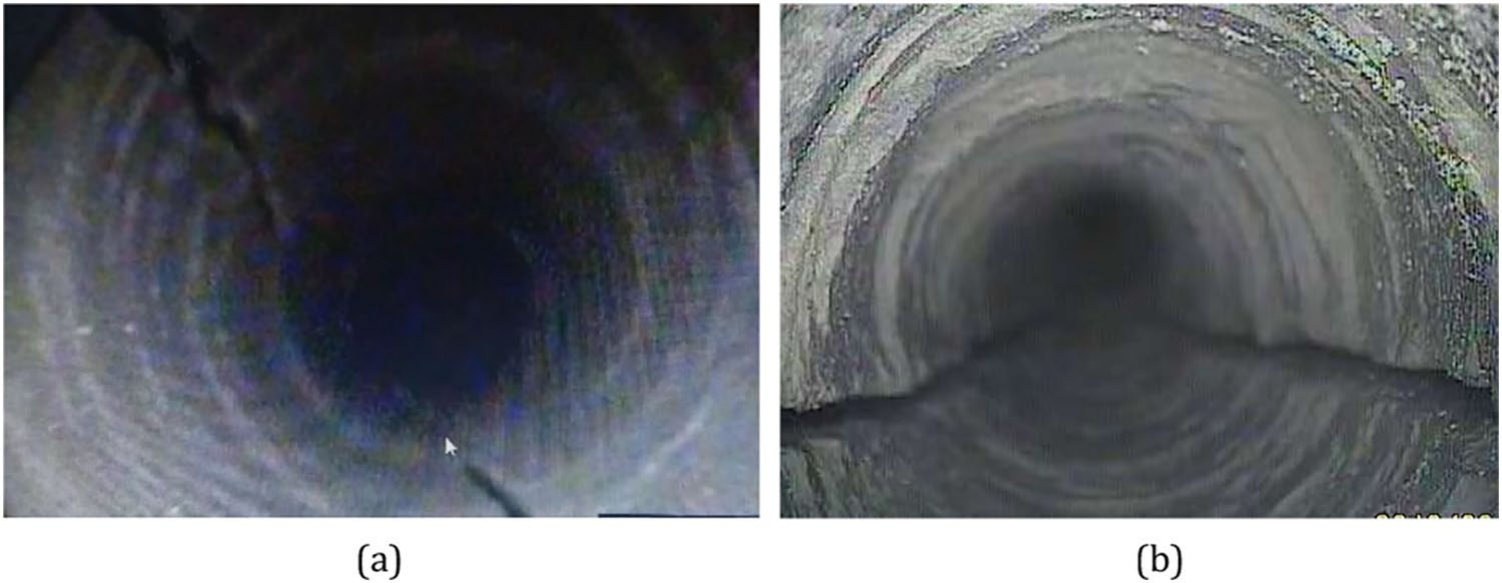
Accumulative blasting effect: (**a**) medium sandstone (Key Stratum I); and (**b**) fine sandstone (Key Stratum II).

By using a borehole viewer, it is possible to observe distinct blast fractures on both sides of the hole, as shown in Fig. 22. Additionally, residual gases that have permeated through from adjacent holes have been observed, indicating that when the hole spacing is 1.0 m, the blast fractures completely penetrate the rock mass between the adjacent blast holes.

**Figure 23:**
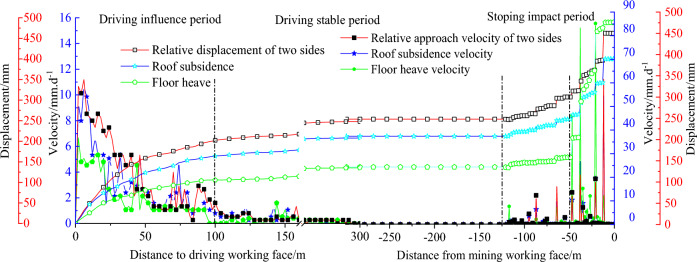
Observation of mine pressure.

As show in Fig. 23, during the tunnelling period: Self-stabilization is achieved in about 10 days, the relative approach maximum velocity of the two sides is 9 mm/d, The maximum velocity of roof subsidence is 11 mm/d, the maximum velocity of floor heave is 7 mm/d, The relative displacement of two sides is 208 mm. The roof subsidence is 248 mm, and the floor heave is 135 mm.

Mining period: The approaching velocities of the roof, floor, and two sides are characterized by intermittent jumping and increasing, with the relative approach maximum velocity of two sides 60 mm/d, the maximum velocity of roof subsidence 63 mm/d, and the maximum velocity of floor heave 84 mm/d.

During the mining period, there were no occurrences of uncontrollable rock deformation and no widespread damage to supporting materials.

## Limitations

1. The successful sliding of composite rock block C_n_ is controlled by factors such as the thickness, length, and width of the Key Stratum after its fracture, but a detailed analysis of these factors is not provided.
2. The cutting length and the cutting angle are two related factors. When the cutting angle decreases and the cutting length remains the same, the cutting height will decrease, which is not elaborated on in this paper.

## Conclusions

1. When the cutting angle is decreased, the deviatoric stress increases, the increase rate: right side (coal pillar) > left side (solid coal) > roof > floor. A slight decrease in the cutting angle, the “inverted trapezoidal” can be placed on the roadway side, preventing the roof from collapsing prematurely, and 75° is selected as the cutting angle.
2. The increase of the cutting length reduces the deviatoric stress, especially when the cutting length (> 20 m) exceeds Key Stratum II, with a significant decrease in deviatoric stress peak value, with characteristics of the right side (coal pillar) > left side (solid coal) > roof > floor.
3. During the process of coal pillar width decreasing from 30 to 4 m, the deviatoric stress distribution of the left side and the floor is in a “single peak” shape, whereas the deviatoric stress distribution of the right side (coal pillar) undergoes a transformation from an asymmetric “double peak” shape to a “bell-shaped”. At the same time, the deviatoric stress distribution of the roof changes from a “single peak” shape to an asymmetric “double peak” shape.
4. Under the same coal pillar width conditions, after roof cutting, the deviatoric stress peak value of the left side (solid coal), right side (coal pillar), and roof decreases significantly, by about 20%, and the peak value of the floor decreases by only a few percent.
5. Accumulative blasting can efficiently complete the artificial cutting of the roof, with a blasting hole spacing not greater than twice the radius of the blasting crack damage, which can be adjusted according to the on-site rock properties.

## Data Availability

All data generated or analysed during this study are included in this published article.
